# Supplementary material for: Endothelial Immunosuppression in Atherosclerosis : Translational Control by Elavl1/HuR
Source: bioRxiv. 2024 Aug 4:2024.08.02.605922. Preprint. [Version 1] doi: 10.1101/2024.08.02.605922 (PMC11312609; doi:10.1101/2024.08.02.605922)
Supplement: Supplement 1 [file NIHPP2024.08.02.605922v1-supplement-1.pdf]

bioRxiv preprint doi: https://doi.org/10.1101/2024.08.02.605922; this version posted August 4, 2024. The copyright holder for this preprint (which was not certified by peer review) is the author/funder, who has granted bioRxiv a license to display the preprint in perpetuity. It is made available under aCC-BY 4.0 International license.

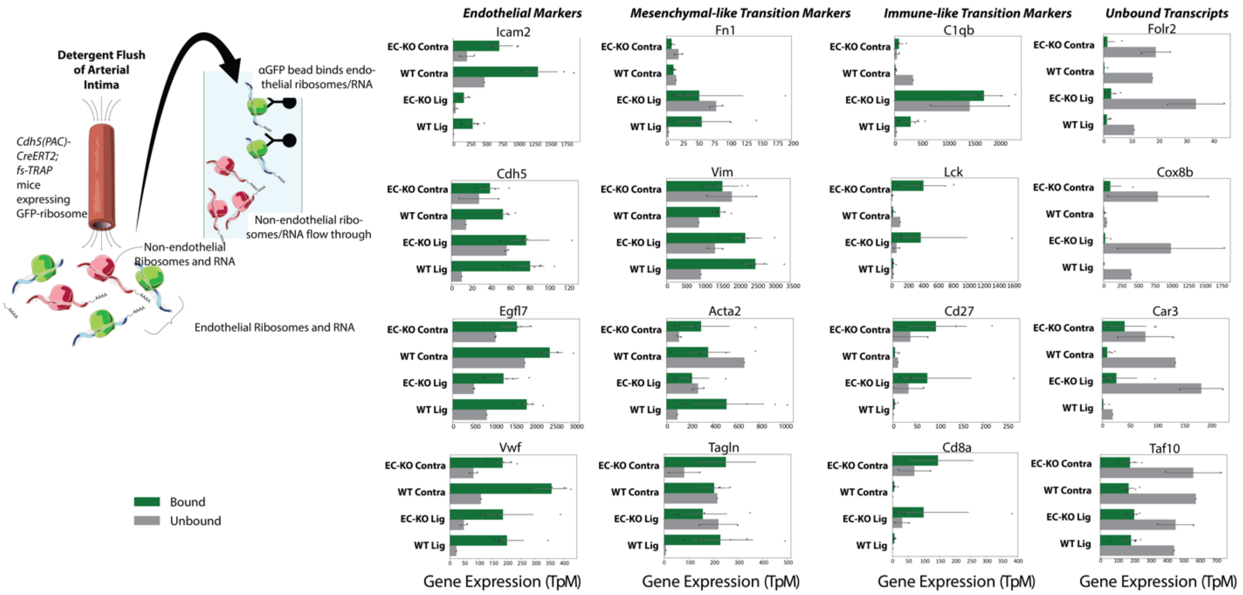

SI Figure 1. Transcript enrichment by ribosomal pulldown (TRAP) from intimal flush. Figure shows the enrichment of individual transcripts over unbound mRNA in the intimal flush. Each point indicates a biological replicate, from the indicated genotype and *in vivo* flow profile, either from the low flow (LDF) or contralateral normal flow (Contra) artery.

bioRxiv preprint doi: https://doi.org/10.1101/2024.08.02.605922; this version posted August 4, 2024. The copyright holder for this preprint (which was not certified by peer review) is the author/funder, who has granted bioRxiv a license to display the preprint in perpetuity. It is made available under aCC-BY 4.0 International license.

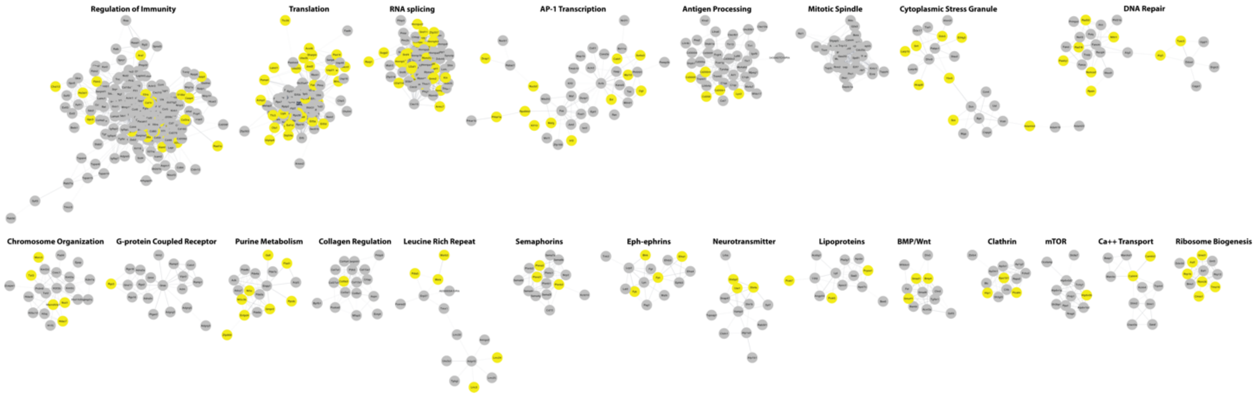

SI Figure 2. Atherosclerosis-associated transcriptional changes in TRAP-mRNA

Cytoscape visualization of all terms associated with low-flow-regulated transcripts in ribosomal mRNA, either by mRNA levels (DESeq2) or splicing (Leafcutter) in TRAP mRNA from endothelial cells in atherosclerotic artery. Expression (gray; padj <0.05) or splicing (yellow; padj <0.05).

bioRxiv preprint doi: https://doi.org/10.1101/2024.08.02.605922; this version posted August 4, 2024. The copyright holder for this preprint (which was not certified by peer review) is the author/funder, who has granted bioRxiv a license to display the preprint in perpetuity. It is made available under aCC-BY 4.0 International license.

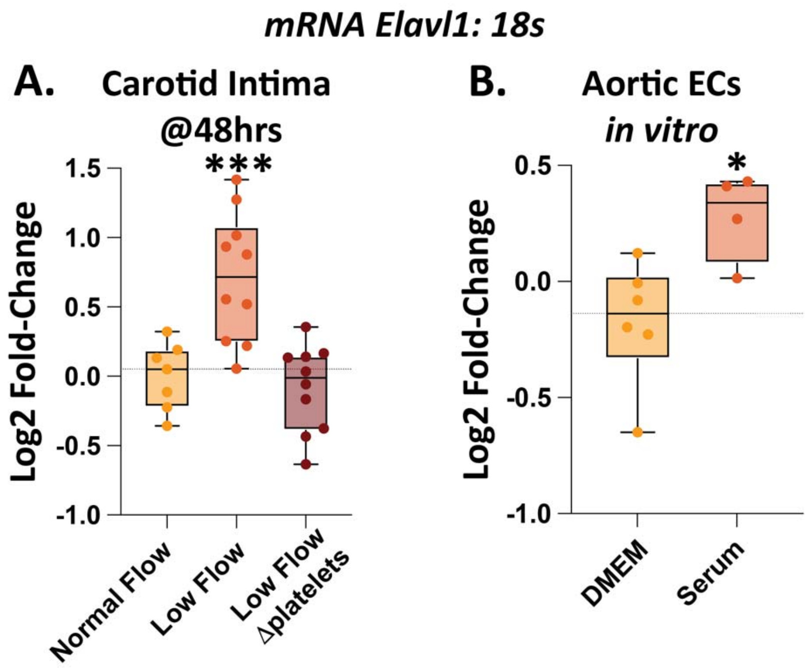

SI Figure 3. Acute changes in Elavl1 expression under atherogenic conditions are platelet dependent. (A) Log2 Fold Change of Elavl1 mRNA expression as detected by qPCR, normalized to housekeeping gene 18s. (B) Isolation of RNA from mouse aortic ECs in culture in the presence or absence of mouse serum, containing platelet releasate. Each point represents one biological replicate well. ***P<0.001, and *P<0.05. ANOVA with post-hoc Dunnett's test, or T-test.

bioRxiv preprint doi: https://doi.org/10.1101/2024.08.02.605922; this version posted August 4, 2024. The copyright holder for this preprint (which was not certified by peer review) is the author/funder, who has granted bioRxiv a license to display the preprint in perpetuity. It is made available under aCC-BY 4.0 International license.

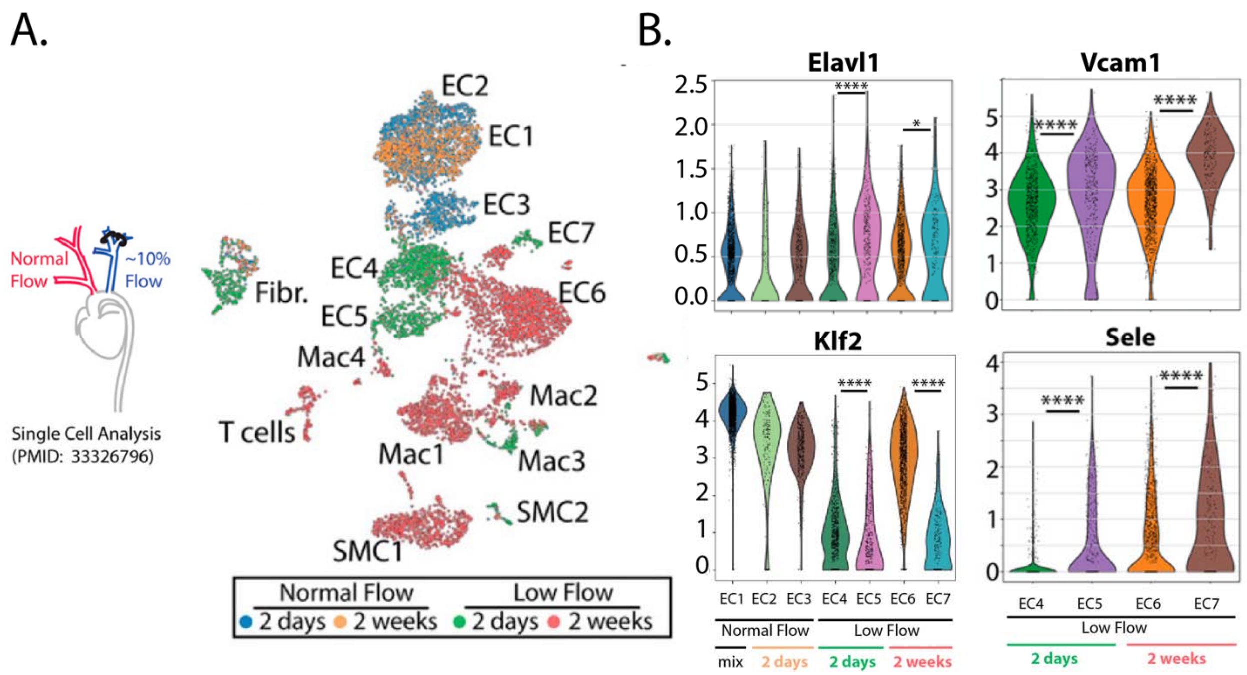

SI Figure 4. Low and disturbed flow-induced Elavl1 expression in mouse endothelium *in vivo*. (A & B) Analysis of single cell data from Andueza, 2020, PMID 33326796. Single cells collected from low flow (LCA) and normal flow (RCA) intima at either 2 days or 2 weeks post-PCAL induction. (A) Leiden clustering of single cell data. ECs subcluster EC1 – EC7. ECs exposed to normal flow cluster together (EC1–EC3) and those exposed to low flow cluster together (EC4–EC7). Subclusters then separate based on length of exposure, low flow at 2 days (EC4–EC5) and low flow at 2 weeks (EC6–EC7). (B) Violin plots of gene expression of Elavl1, flow-responsive Klf2, and EC inflammatory genes Icam1 and Vcam1. (B) ****p<0.0001, by Wilcoxon rank test.

bioRxiv preprint doi: https://doi.org/10.1101/2024.08.02.605922; this version posted August 4, 2024. The copyright holder for this preprint (which was not certified by peer review) is the author/funder, who has granted bioRxiv a license to display the preprint in perpetuity. It is made available under aCC-BY 4.0 International license.

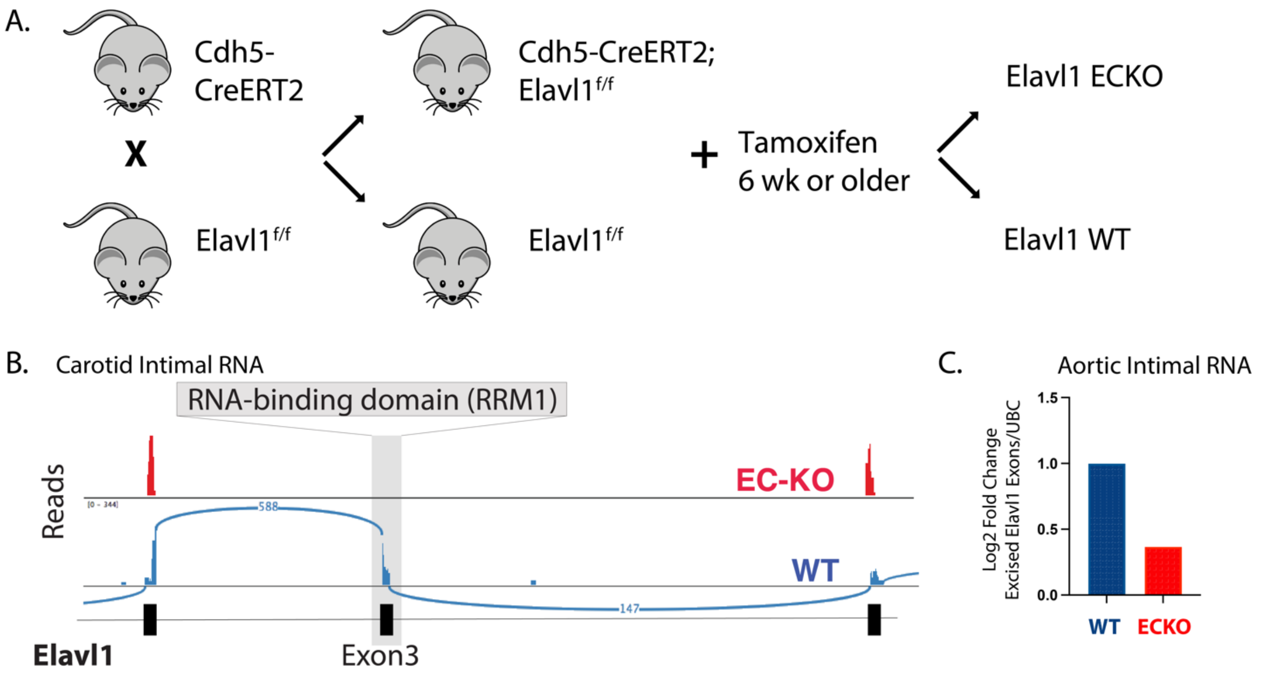

SI Figure 5. Tamoxifen-induced recombination of *Cdh5-CreERT2; Elavl1f/f* and *Elavl1f/f* littermate controls decreased levels of excised region of Elavl1 mRNA. (A) Schematic of generation of experimental mice. (B) Representative RNA reads from an example littermate pair from intimal EC trizol flush. (C) RNA expression levels of excised Elavl1 exons from FACS-isolated mAECs detected by qPCR, normalized to UBC.

bioRxiv preprint doi: https://doi.org/10.1101/2024.08.02.605922; this version posted August 4, 2024. The copyright holder for this preprint (which was not certified by peer review) is the author/funder, who has granted bioRxiv a license to display the preprint in perpetuity. It is made available under aCC-BY 4.0 International license.

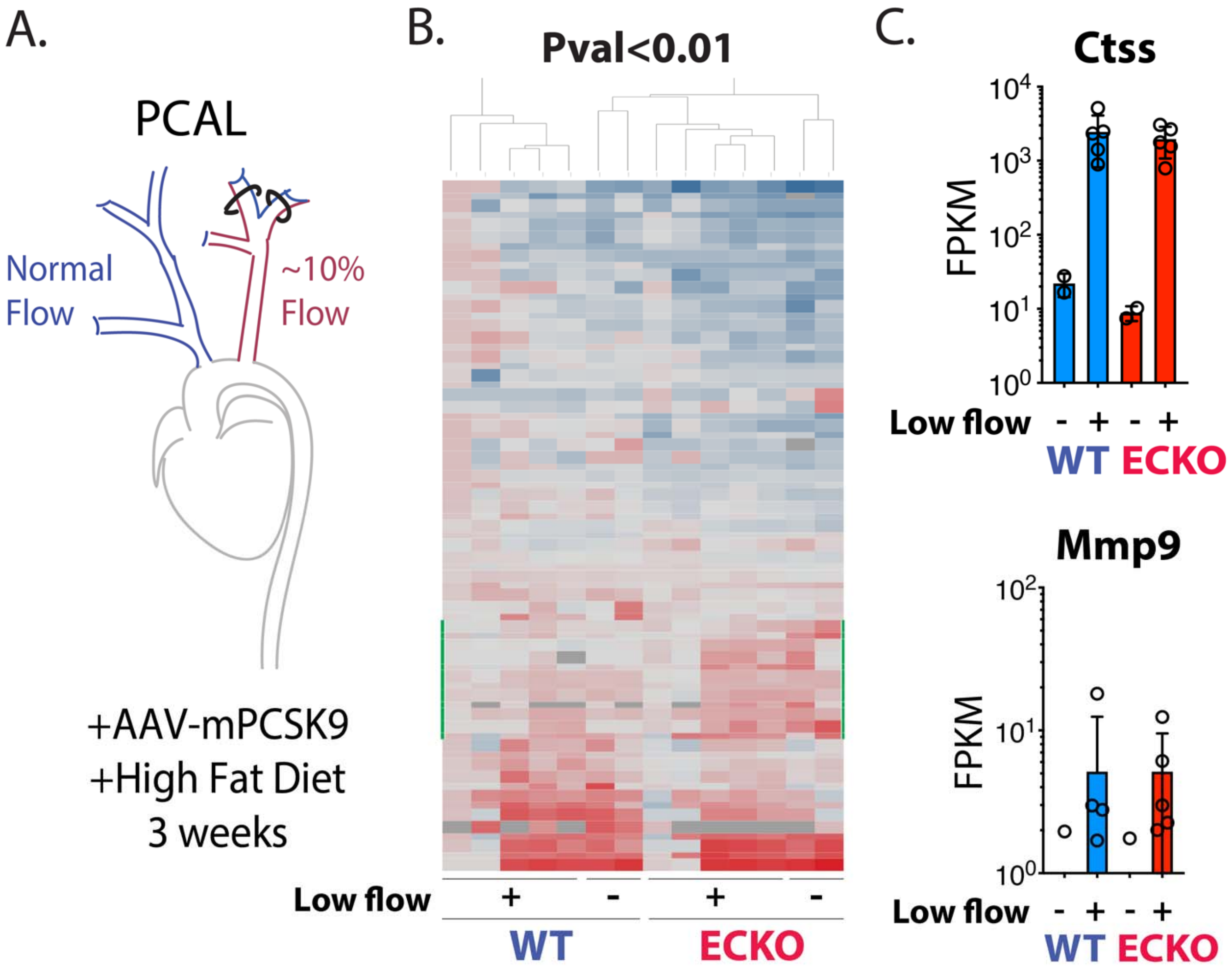

SI Figure 6. Elavl1 deletion leads few changes in canonically 3'utr stabilized transcripts in bulk endothelial transcriptome. (A) Schematic of PCAL model. Total intimal EC RNA was isolated from low flow LCA and normal flow RCA after three weeks of PCAL and hyperlipidemia via trizol flush. (B&C) Quantification of transcript levels from arterial intima of WT or Elavl1 ECKO mice. Analysis by RSEM and DESeq2 (B) Heat map showing the expression levels of genes for all transcripts achieving P value <0.01. (C) Expression levels of Elavl1 canonically stabilized targets, Ctss and Mmp9. (B&C) N=5 and 5 biological replicates of Elavl1 ECKO and WT mice under low flow, N=2 and 2 under normal flow.

bioRxiv preprint doi: https://doi.org/10.1101/2024.08.02.605922; this version posted August 4, 2024. The copyright holder for this preprint (which was not certified by peer review) is the author/funder, who has granted bioRxiv a license to display the preprint in perpetuity. It is made available under aCC-BY 4.0 International license.

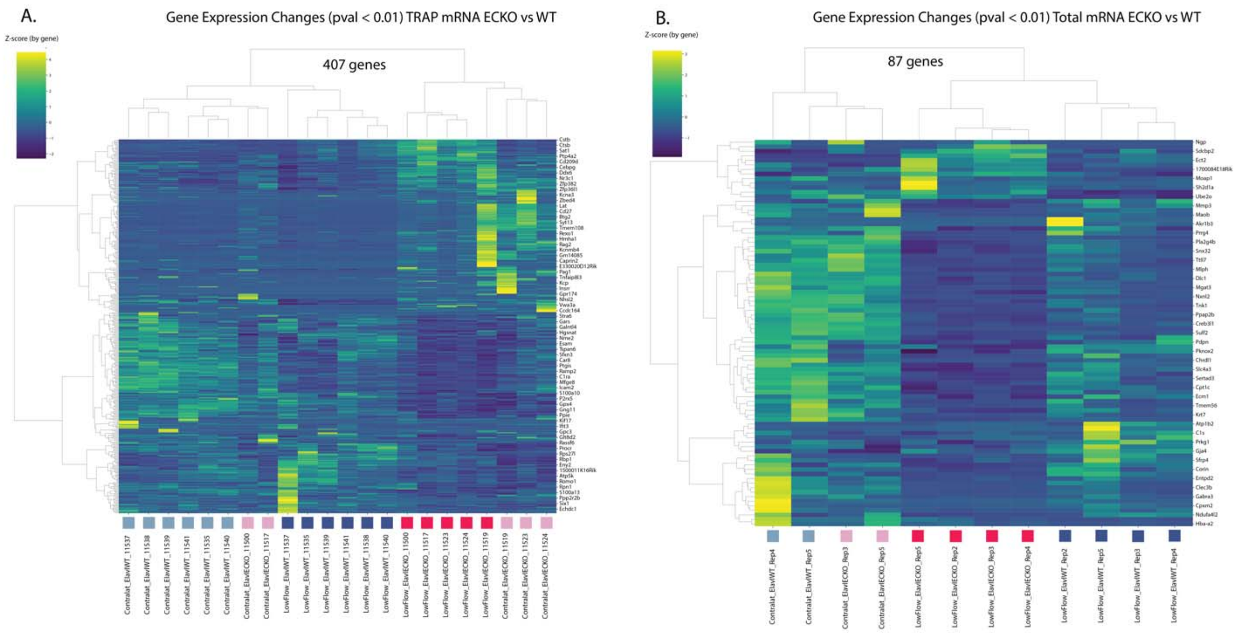

SI Figure 7. Effects of Elavl1 deletion on TRAP and Total mRNA. Figure shows the transcripts differentially regulated (unadjusted P<0.01) in ribosome-associated RNA (A) and total RNA (B). Mice from which the RNA was isolated are indicated below, with coloring to indicate the genotype (blue = WT and red = ECKO), and shading to indicate the flow pattern (dark = low flow, ligated and light = normal flow, contralateral).

bioRxiv preprint doi: https://doi.org/10.1101/2024.08.02.605922; this version posted August 4, 2024. The copyright holder for this preprint (which was not certified by peer review) is the author/funder, who has granted bioRxiv a license to display the preprint in perpetuity. It is made available under aCC-BY 4.0 International license.

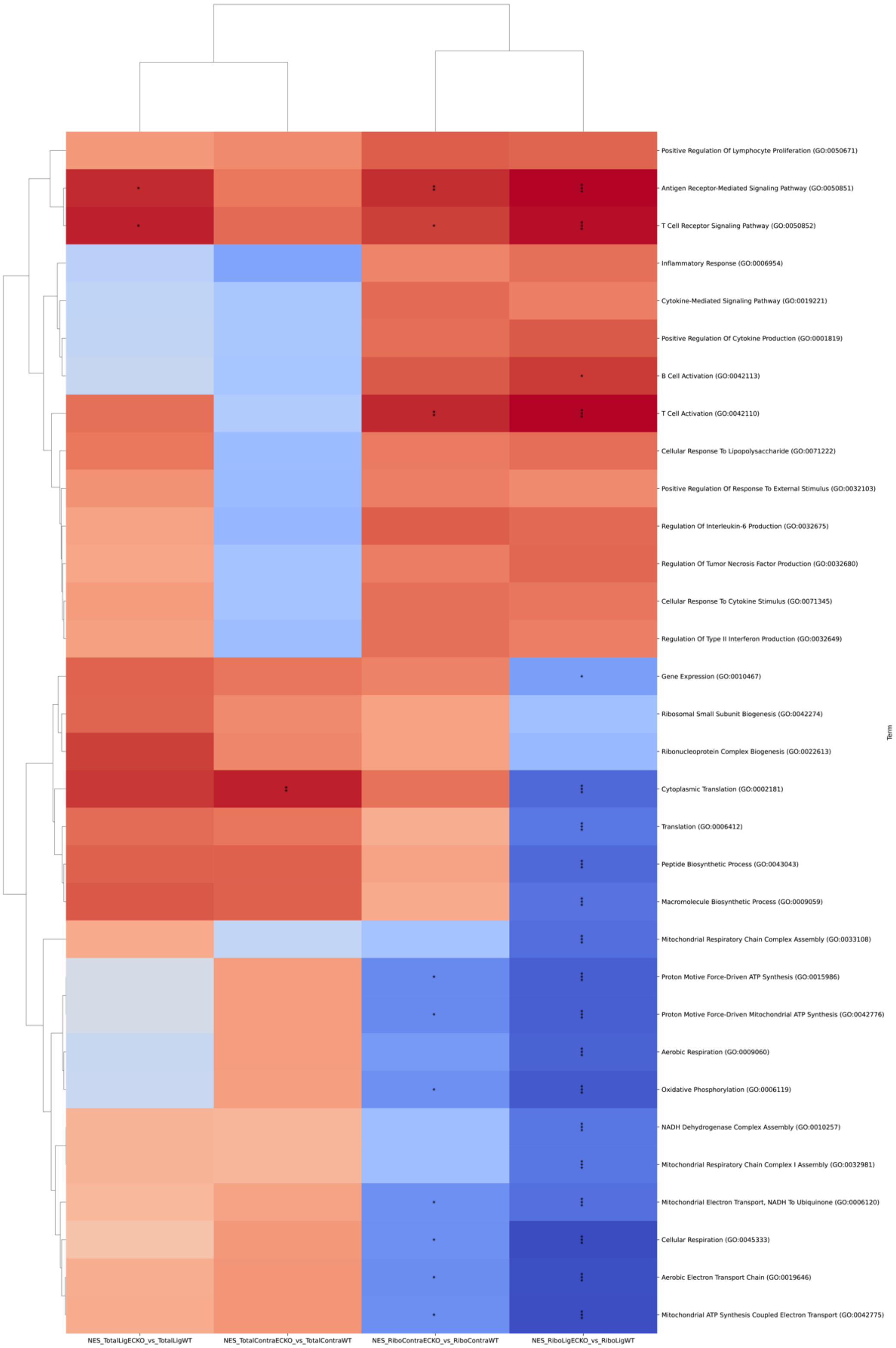

SI Figure 8. GSEA Terms among transcripts regulated differentially between ECKO and WT mice. Companion figure to main Figure 2J, showing the individual terms different between each response (GSEA weight 1). Plotted colors with the same Normalized Enrichment Score (NES) scale shown in main Figure 2J. *False Discovery Rate (FDR)<0.05, **FDR<0.01, ***FDR<0.001.

bioRxiv preprint doi: https://doi.org/10.1101/2024.08.02.605922; this version posted August 4, 2024. The copyright holder for this preprint (which was not certified by peer review) is the author/funder, who has granted bioRxiv a license to display the preprint in perpetuity. It is made available under aCC-BY 4.0 International license.

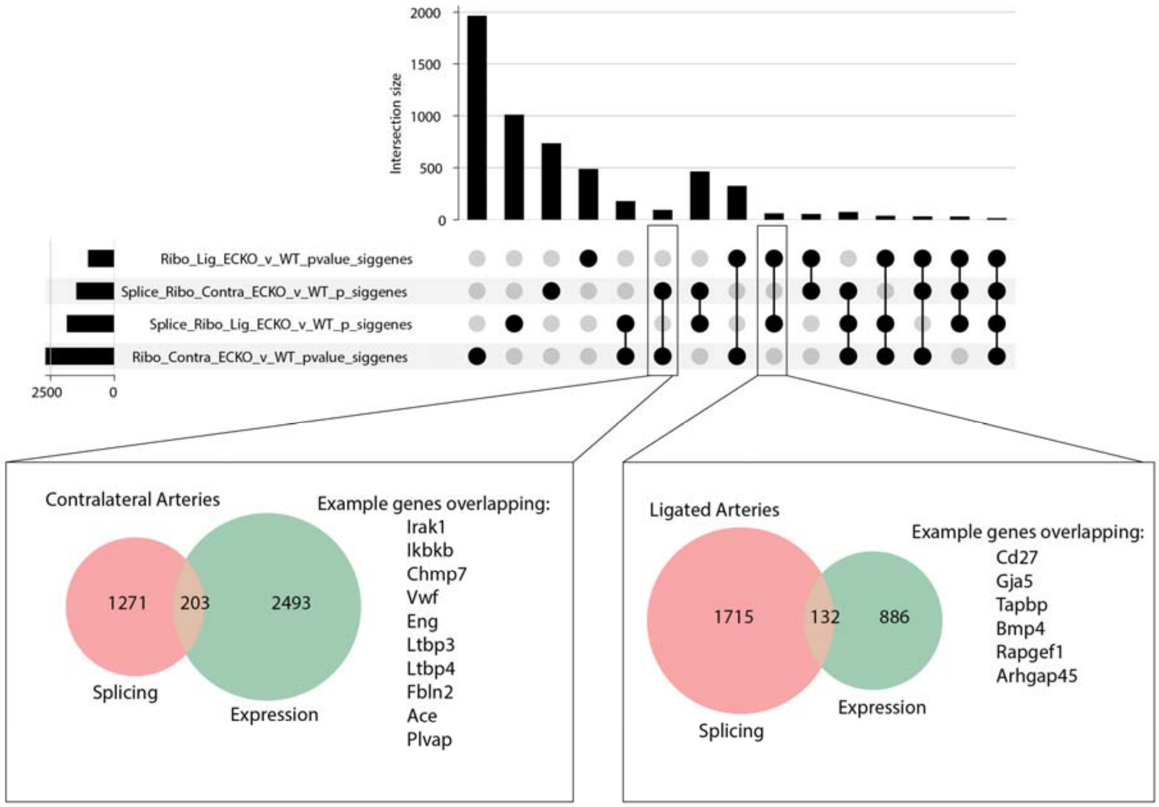

SI Figure 9.

Overlap between genes regulated by splicing or by expression in ECKO mice.
Upset plot and Venn diagram of specific examples, showing the overlap between genes regulated by splicing (Leafcutter, $p<0.05$) or by level at the ribosome (DESeq2, $p<0.05$). Example genes in overlap are shown adjacent to the Venn diagram.

bioRxiv preprint doi: https://doi.org/10.1101/2024.08.02.605922; this version posted August 4, 2024. The copyright holder for this preprint (which was not certified by peer review) is the author/funder, who has granted bioRxiv a license to display the preprint in perpetuity. It is made available under aCC-BY 4.0 International license.

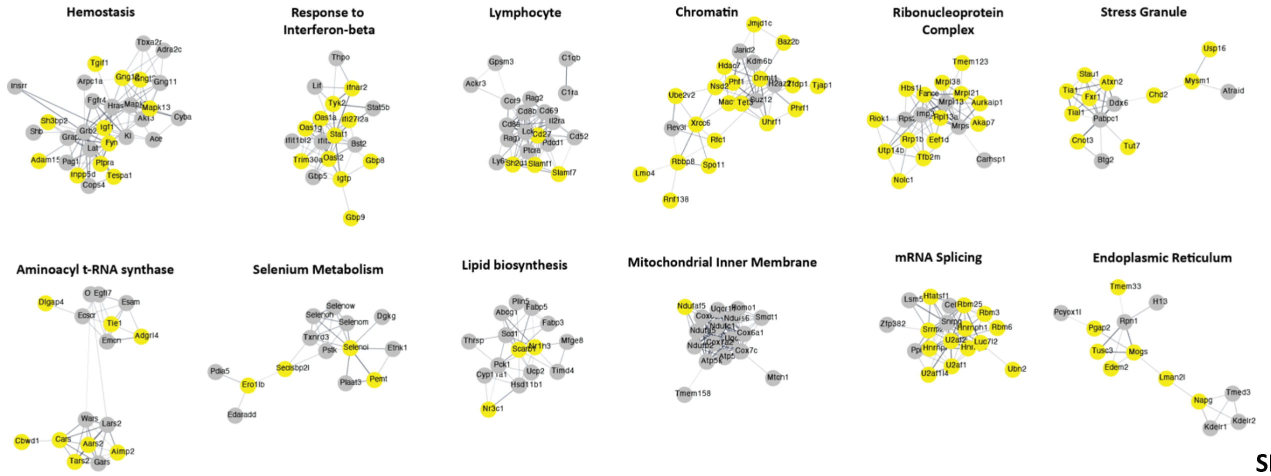

SI Figure 10.

Pathways enriched among TRAP-mRNA changes associated with loss of Elavl1 in low flow endothelium. Cytoscape visualization of all terms associated with low flow transcripts regulated by Elavl1 ECKO in ribosomal mRNA, either by expression (gray; padj <0.05) or splicing (yellow; padj <0.05).

bioRxiv preprint doi: https://doi.org/10.1101/2024.08.02.605922; this version posted August 4, 2024. The copyright holder for this preprint (which was not certified by peer review) is the author/funder, who has granted bioRxiv a license to display the preprint in perpetuity. It is made available under aCC-BY 4.0 International license.

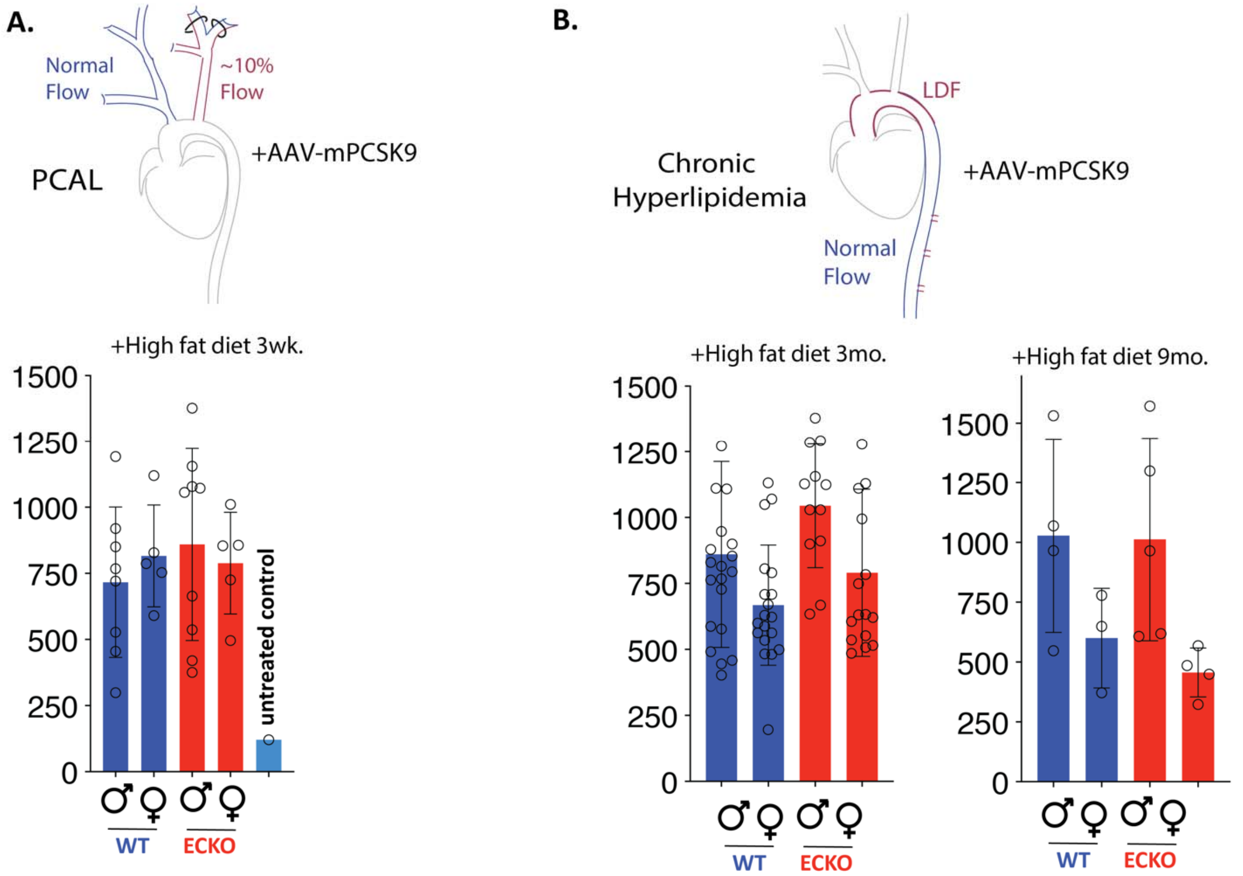

SI Figure 11.

Serum lipid levels are similar in ECKO and WT controls. (A&B) Blood serum total cholesterol levels (mg/dL) at time of collection for PCAL and chronic plaque model, separated by sex and WT vs Elavl1 ECKO.

bioRxiv preprint doi: https://doi.org/10.1101/2024.08.02.605922; this version posted August 4, 2024. The copyright holder for this preprint (which was not certified by peer review) is the author/funder, who has granted bioRxiv a license to display the preprint in perpetuity. It is made available under aCC-BY 4.0 International license.

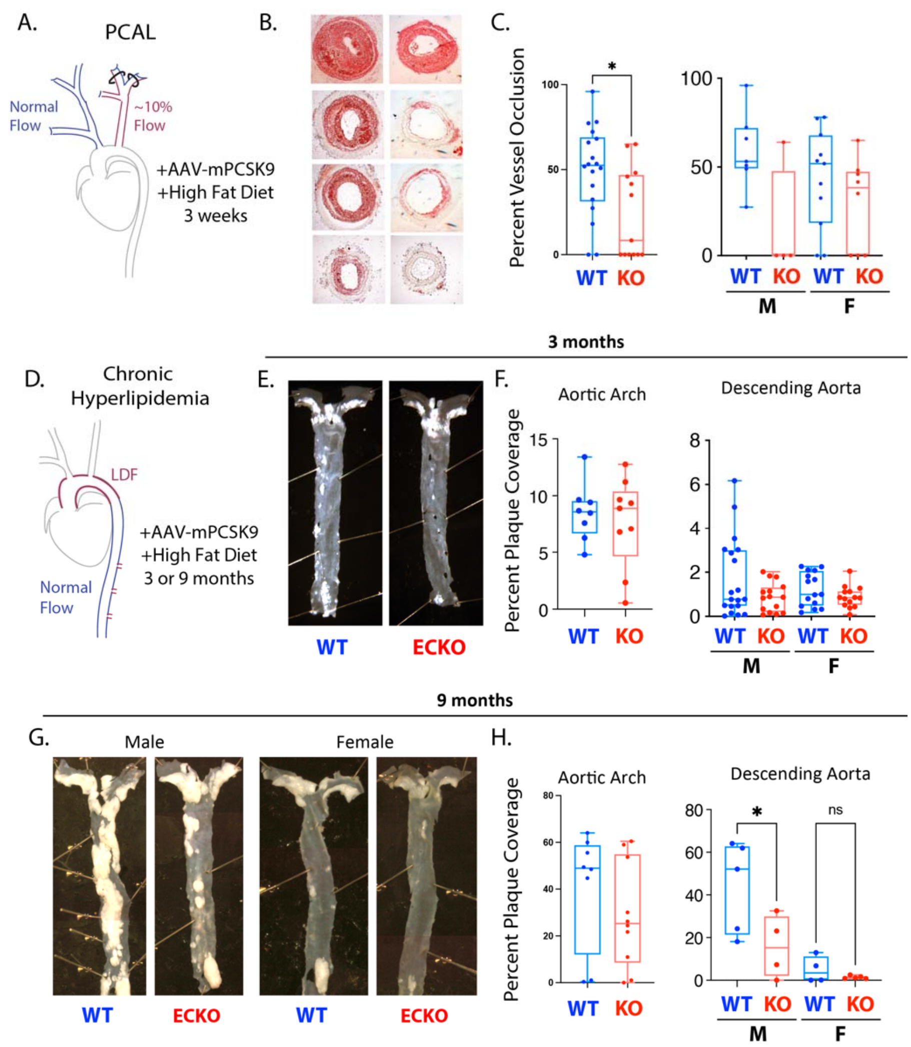

SI Figure 12. Plaque development in PCAL and at sites of disturbed flow in chronic hyperlipidemia is reduced in Elavl1 ECKO mice. Analysis of plaque coverage by sex in PCAL and chronic plaque development (3 mo and 9 mo hyperlipidemia). (A–C) analysis of percent carotid occlusion at 3 weeks after low flow and hyperlipidemia, with images stained by OilRedO (B) and quantitation (C). (A–H) Similar analysis of *en face* plaque in chronic response at 3 mo. (D–F) and 9 mo. (G–H).

bioRxiv preprint doi: https://doi.org/10.1101/2024.08.02.605922; this version posted August 4, 2024. The copyright holder for this preprint (which was not certified by peer review) is the author/funder, who has granted bioRxiv a license to display the preprint in perpetuity. It is made available under aCC-BY 4.0 International license.

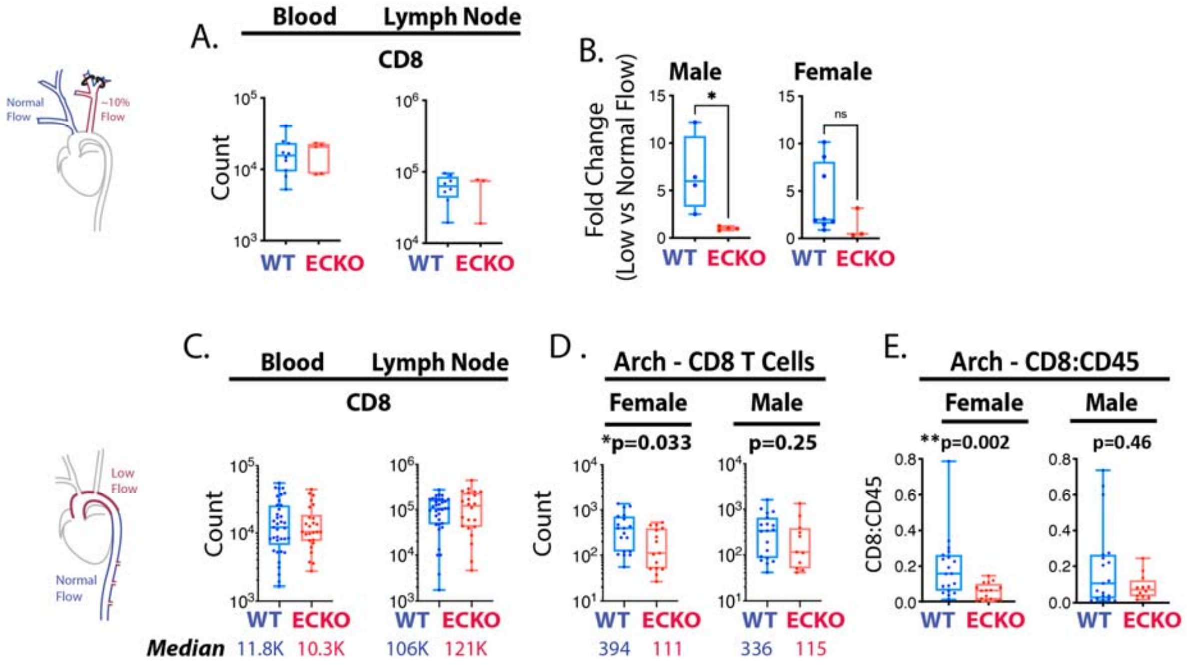

SI Figure 13. Elavl1 ECKO reduces plaque CD8 T cells without substantial effects on blood or lymphnode levels. (A–E) Quantification of flow cytometry analysis. (A) Absolute count of CD8 T cells in blood and lymph node in mice with PCAL. (B) Ratio of CD8 T cells in low flow atherogenic artery relative to contralateral control artery, separated by sex. (C-D) Absolute count of CD8 T cells with 3 months of hyperlipidemia in blood and lymph node in mice (C) and aortic arch (D). (E) Ratio of CD8:CD45 in aortic arch in female and male mice. (A) N=12 WT, N=7 EC-KO (B) N=8 Female WT, N=4 Male WT; N=3 Female ECKO, N=4 Male ECKO. (C&D) N=37 WT, N=26 ECKO. (E) N=19 Female WT, N=19 Male WT; N=15 Female ECKO, N=11 Male ECKO. Comparisons by two-tailed Mann-Whitney test.

bioRxiv preprint doi: https://doi.org/10.1101/2024.08.02.605922; this version posted August 4, 2024. The copyright holder for this preprint (which was not certified by peer review) is the author/funder, who has granted bioRxiv a license to display the preprint in perpetuity. It is made available under aCC-BY 4.0 International license.

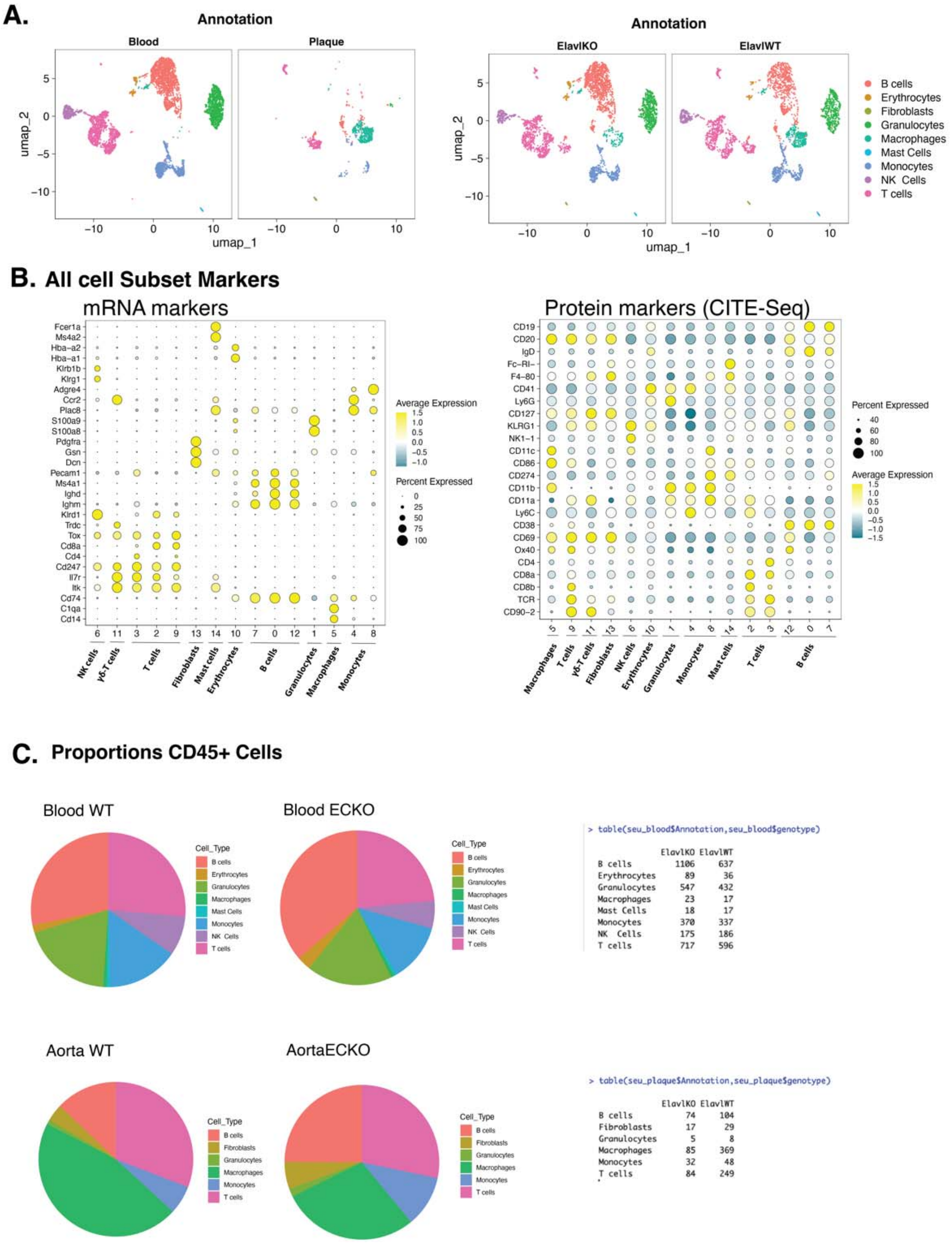

SI Figure 14. CITE-seq analysis of hematopeotic cells in blood and established aortic plaque of ECKO mice. Graphs showing data analysis of 10x CITE-seq from blood and CD45+ cells from aorta, hashtagged in one 10x reaction. Equal amounts of red-blood cell lysed blood samples were included, and all of the CD45+ aortic cells from each aorta were combined. Ratios between blood samples and between plaque sample therefore reflect *in vivo* ratios. (A) UMAP separated by tissue source and (B) genotype. (B) Dot plot showing the expression of top RNA and protein markers in hematopoietic subsets. (C) Pie charts showing the percent contribution and total numbers of each cell type in blood and aortic tissue.

bioRxiv preprint doi: https://doi.org/10.1101/2024.08.02.605922; this version posted August 4, 2024. The copyright holder for this preprint (which was not certified by peer review) is the author/funder, who has granted bioRxiv a license to display the preprint in perpetuity. It is made available under aCC-BY 4.0 International license.

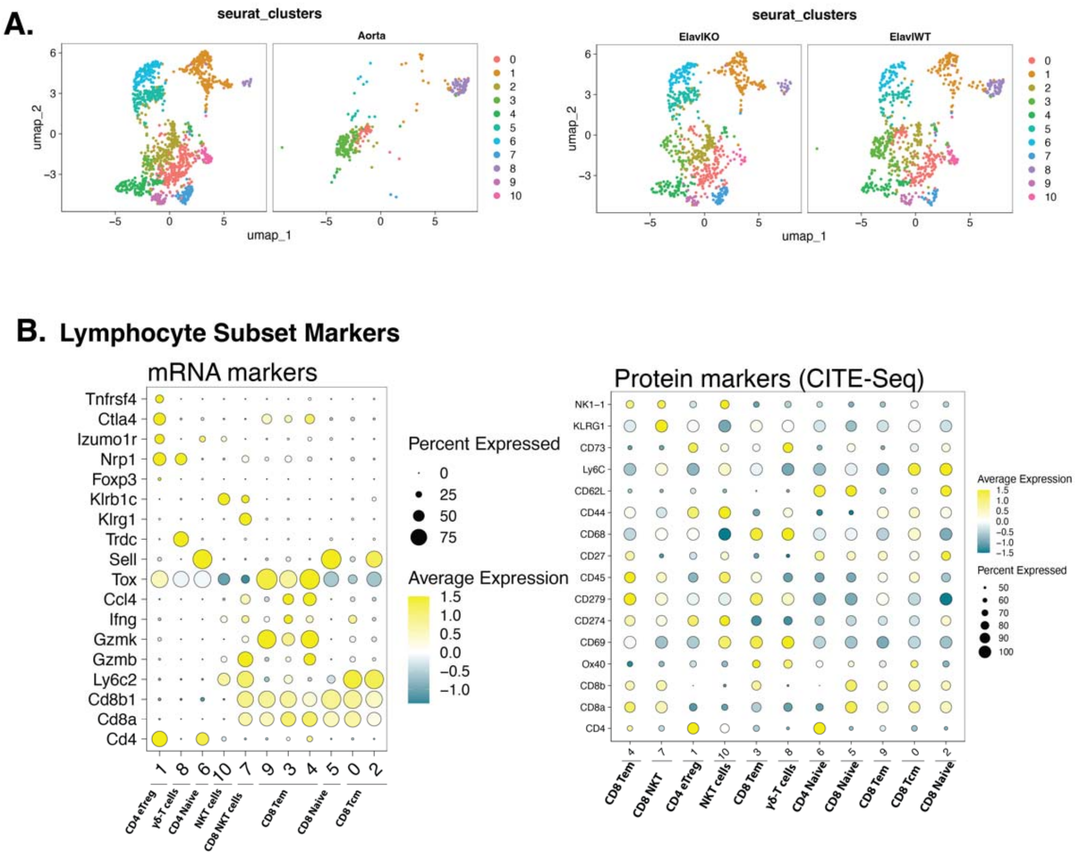

C. Proportions Lymphocytes

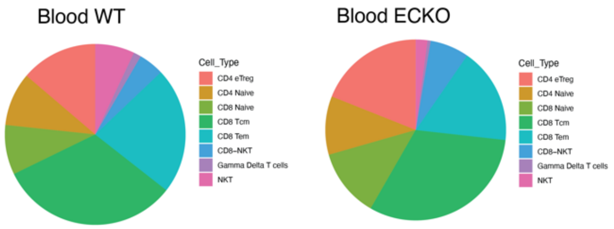


```
> table(seu_lymph_blood$Annotation,seu_lymph_blood$genotype)

                    ElavlKO ElavlWT
  CD4 eTreg             136      82
  CD4 Naive              75      57
  CD8 Naive              88      53
  CD8 Tcm               226     192
  CD8 Tem               123     135
  CD8-NKT                50      27
  Gamma Delta T cells     4       8
  NKT                    15      42
```

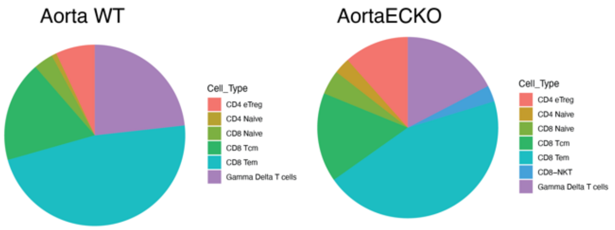


```
> table(seu_lymph_plaque$Annotation,seu_lymph_plaque$genotype)

                    ElavlKO ElavlWT
  CD4 eTreg               8      16
  CD4 Naive               2       2
  CD8 Naive               3       8
  CD8 Tcm                11      41
  CD8 Tem                31     108
  CD8-NKT                 2       0
  Gamma Delta T cells    12      53
```

SI Figure 15. CITE-seq analysis of lymphocytes in blood and established aortic plaque of ECKO mice. Graphs showing data analysis of 10x CITE-seq from blood and CD45+ cells from aorta, hashtagged in one 10x reaction. Equal amounts of red-blood-cell-lysed blood samples were included, and all of the CD45+ aortic cells from each aorta were combined. Ratios between blood samples and between plaque sample therefore reflect *in vivo* ratios. (A) UMAP separated by tissue source and (B) genotype. (B) Dot plot showing the expression of top RNA and protein markers in lymphocyte subsets. (C) Pie charts showing the percent contribution and total numbers of each cell type in blood and aortic tissue.

bioRxiv preprint doi: https://doi.org/10.1101/2024.08.02.605922; this version posted August 4, 2024. The copyright holder for this preprint (which was not certified by peer review) is the author/funder, who has granted bioRxiv a license to display the preprint in perpetuity. It is made available under aCC-BY 4.0 International license.

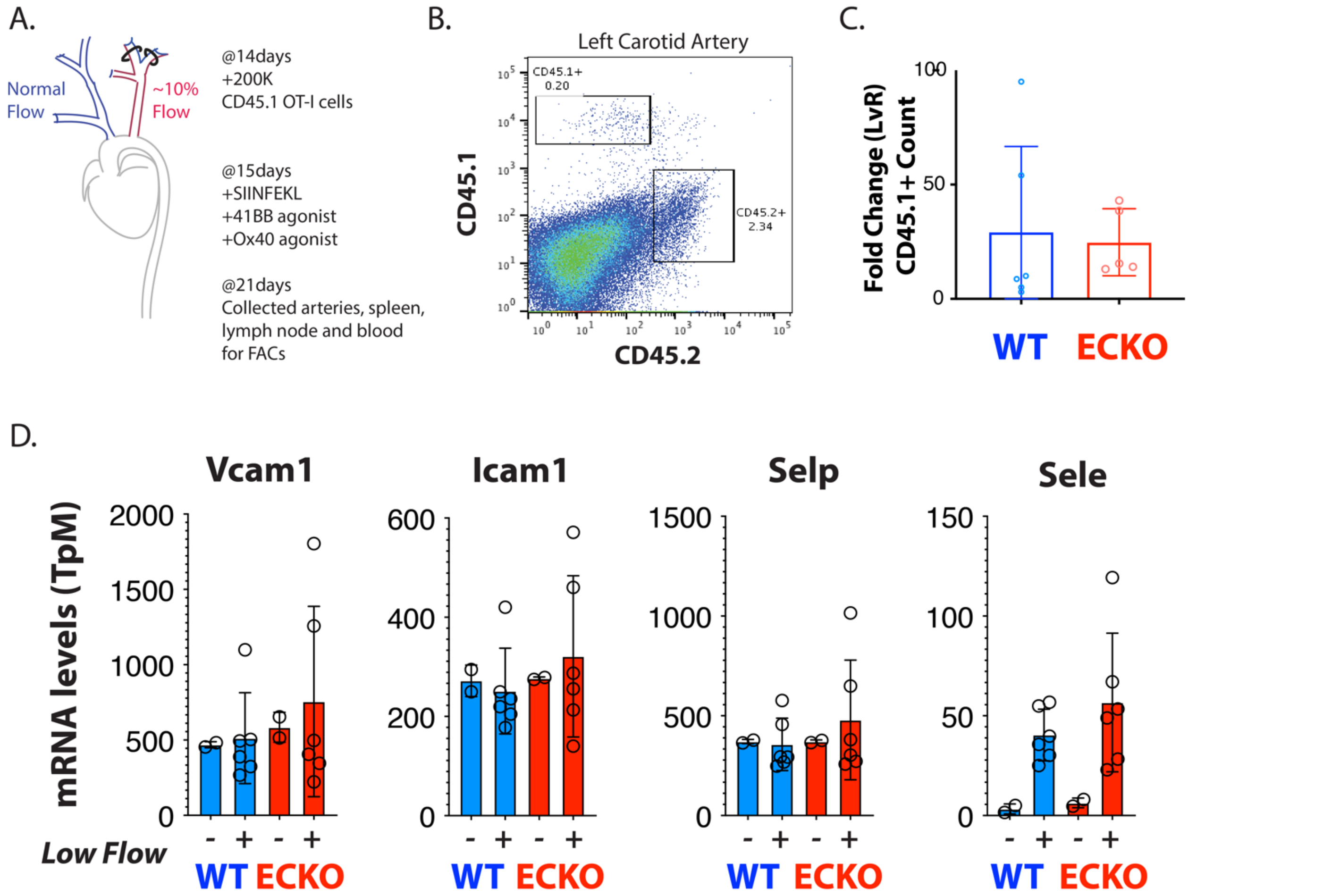

SI Figure 16. EC Elavl1 does not affect activated CD8+ T cell recruitment to atherosclerotic plaque or expression of adhesion molecules. (A) Schematic of adoptive transfer experimental design. (B) Representative flow cytometry plot featuring detection of both endogenous (CD45.2+) immune cells and adoptively transferred CD45.1+ immune cells in the LCA. (C) Quantification of fold change of CD45.1 numbers recruited to LDF flow LCA over normal flow RCA. (D) mRNA expression (transcript per million) levels of canonical immune cell recruitment molecules in Elavl1 ECKO vs WT in total intimal EC RNAseq data.

bioRxiv preprint doi: https://doi.org/10.1101/2024.08.02.605922; this version posted August 4, 2024. The copyright holder for this preprint (which was not certified by peer review) is the author/funder, who has granted bioRxiv a license to display the preprint in perpetuity. It is made available under aCC-BY 4.0 International license.

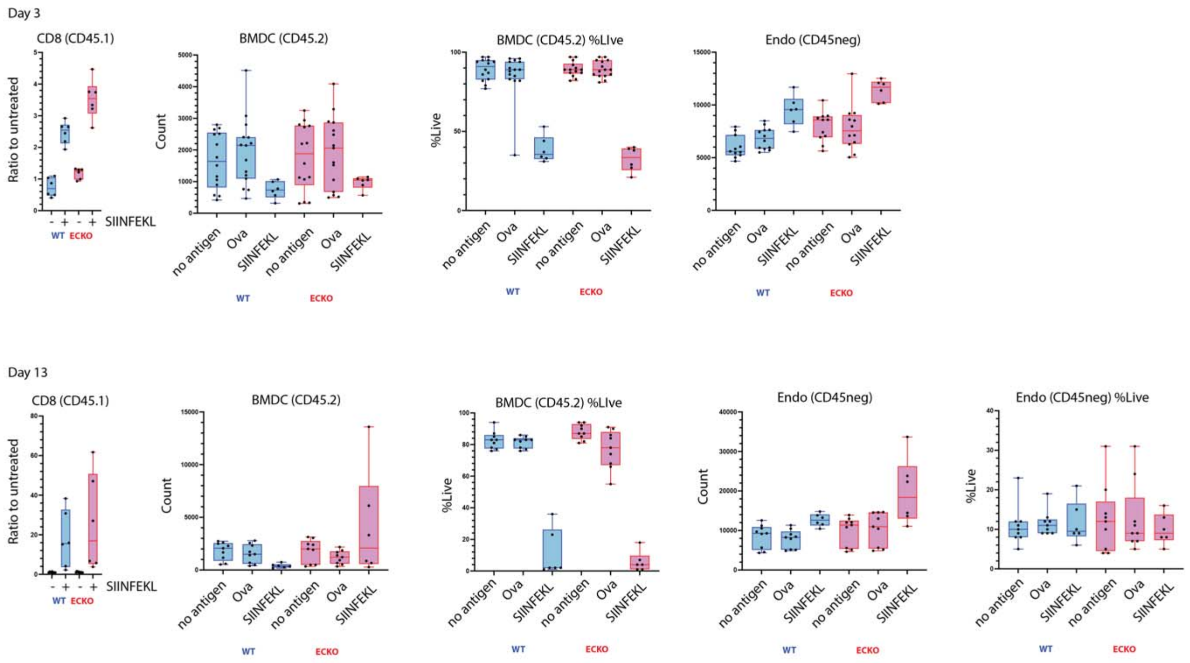

SI Figure 17. Bone marrow derived dendritic (BMDCs) and endothelial cell numbers are not significantly changed by the loss of Elavl1 *in vitro*. Graphs show the quantitation of cell numbers of CD45.2+ bone marrow derived dendritic cells, and CD45 negative cells (mainly endothelial, but also including T cells or BMDCs which lost CD45). The BMDCs were either pulsed with SIINFEKL antigen before the addition of T cells (D0), or supplemented with full-length ovalbumin for the indicated period of time (D0 to D3 or D0 to D13). Quantitation is by flow-cytometry, and each point represents cell count from one well of a 96-well plate across multiple individual experimental replicates.
